# Administration of Natural Fish and Algal Oils in Nanoparticle Form to Pregnant Gilts and Newborn Piglets: Biochemical Effects and Spatial–Socio-Economic Implications for Regional Food Systems

**DOI:** 10.3390/ijms26189158

**Published:** 2025-09-19

**Authors:** Paweł Kowalczyk, Monika Sobol, Joanna Makulska, Andrzej Węglarz, Apoloniusz Kurylczyk, Grzegorz Skiba

**Affiliations:** 1 Department of Animal Nutrition, The Kielanowski Institute of Animal Physiology and Nutrition, Polish Academy of Sciences, Instytucka 3, 05-110 Jabłonna, Poland; m.sobol@ifzz.pl; 2 Department of Genetics, Breeding and Animal Ethology, Hugo Kołłątaj University of Agriculture in Krakow, Ave. A. Mickiewicza 21, 31-120 Kraków, Poland; joanna.makulska@urk.edu.pl (J.M.); andrzej.weglarz@urk.edu.pl (A.W.); 3 Institute of Spatial Management and Socio-Economic Geography, University of Szczecin, ul. Papieża Jana Pawłą II 22a, 70-453 Szczecin, Poland; apoloniusz.kurylczyk@usz.edu.pl

**Keywords:** maternal programming, fish and algal oil nanoparticles, newborn piglets, oxidative stress, antioxidant defence system, spatial diffusion of nutritional innovation

## Abstract

This study investigated the influence of long-chain n-3 polyunsaturated fatty acids (PUFAs) on the activity of antioxidant defence systems and DNA repair enzymes in the liver of newborn piglets born to gilts that were supplemented with fish oil or algal oil during pregnancy. The oils were offered in their natural form or as nanoparticles. Daily doses of both natural and nano-encapsulated oils were calculated to provide each gilt with 3100 mg of docosahexaenoic acid (DHA; 600 mg for the gilt and 250 mg for each foetus). Liver samples were collected from six piglets per gilt within 24 h after birth. Activities of superoxide dismutase (SOD), catalase (CAT), and glutathione peroxidase (GPx) were measured spectrophotometrically, while DNA repair enzyme activities—formamidopyrimidine-DNA glycosylase (FPG), thymine-DNA glycosylase (TDG), and N-methylpurine DNA glycosylase (MPG)—were assessed by Fpg protein digestion. SOD activity was lowest in piglets from gilts supplemented with algal oil, fish oil, and nano-encapsulated fish oil. Piglets born to the gilts that received algal oil nanoparticles showed higher activity (1.57 U/mg), while the highest activity was recorded in control piglets. CAT activity followed a similar trend; it was lowest in algal oil-supplemented mothers and highest in controls. GPx activity was lowest in piglets born to gilts that received algal oil (both forms) and highest in controls. The FPG activity in piglets birthed by PUFA-supplemented gilts was approximately half that of MPG and TDG, indicating reduced oxidative DNA damage. Both fish oil and algal oil, regardless of the form administered, effectively reduce oxidative stress in pregnant gilts and the associated DNA damage in the livers of their offspring. These findings suggest that maternal supplementation with long-chain n-3 PUFAs can protect newborn piglets from oxidative damage. Furthermore, regional disparities in access to functional foods underline the importance of targeted strategies that integrate local food systems and health planning to promote nutritional equity.

## 1. Introduction

Animal production exposes livestock to various stressors, including physiological (rapid growth rate, high meat yield), environmental (parturition and weaning, air quality, temperature, and stocking density) and nutritional (feed quality and availability) factors. At the molecular level, stress is associated with the overproduction of reactive oxygen species (ROS), oxidative stress, and disturbances in redox balance [1,2]. ROS are generated during normal cellular metabolism but may also arise from pathological processes and extracellular sources. Oxidative stress occurs when ROS generation and cellular respiration by-products exceed the capacity of local antioxidant defences, causing damage to cellular components [3]. Antioxidant defence mechanisms consist of overlapping chemical reactions, in which the product of one reaction is the substrate for the next. Recent studies [4] have identified several lines of antioxidant defence against oxidative stress. The first and most important line comprises endogenous enzymes, including superoxide dismutases (SODs), catalase (CAT), and glutathione peroxidase (GPx), which remove hydrogen peroxide and prevent the formation of reactive oxygen species (ROS). These enzymes neutralise molecules with the potential to form free radicals or produce secondary radicals [5]. Although some authors [6] consider the synthesis of glutathione (GSH) and thioredoxin (Trx-Red or Trx-SH) a second line of antioxidant defence, because these molecules act as cofactors for antioxidant enzymes, other researchers [4] include GSH and Trx-Red/Trx-SH in the first line. The second line involves low-molecular-weight antioxidants (vitamin C, vitamin E, carotenoids, and flavonoids), often referred to as scavenging antioxidants, which capture active radicals to inhibit chain initiation and break chain propagation reactions. The third line of antioxidant defence comprises enzymes that are activated following free radical-induced damage, repairing DNA, proteins and lipids affected by oxidative stress. They identify, break down, and eliminate oxidised or damaged proteins, DNA, and lipids, preventing the accumulation of toxic molecules [5]. Some authors distinguish a fourth line of antioxidant defence [7,8], which functions through adaptive mechanisms. This system utilises signals generated by free radical production to induce the synthesis and transport of appropriate antioxidants to the site of action to prevent free radical formation or activity.

According to Halliwell [9], the reaction rates at which ROS damage DNA, proteins, and membrane lipids are significantly greater than those involving low-molecular-weight antioxidants, which limits the radical-scavenging capacity of the latter. Endogenous enzymes of the first line of antioxidant defence are therefore the most effective means of preventing oxidative stress and related cellular damage. SOD is the primary detoxification enzyme and the most potent cellular antioxidant, protecting body cells from excessive oxygen radicals, free radicals, and other harmful agents that promote ageing or cell death [10]. It catalyses the dismutation of two superoxide anions (*O_2_) to hydrogen peroxide (H_2_O_2_) and molecular oxygen (O_2_), neutralising the harmful superoxide anion [5]. As a metalloenzyme, SOD requires a metal cofactor for activation, and its isoforms depend on the type of metal ion used [11]. CAT is another ubiquitous antioxidant enzyme, present in almost all oxygen-utilising tissues, that employs iron or manganese as cofactors and catalyses the conversion of H_2_O_2_ to water and oxygen, completing the detoxification initiated by SOD [12]. CAT is abundant, and highly efficient at locating H_2_O_2_ molecules, primarily in the peroxisomes, but is absent in mammalian mitochondria. GPx also reduces H_2_O_2_ to water and converts lipid peroxides to corresponding alcohols, mainly in mitochondria [13]. GPx requires selenium as a cofactor and is often classified as a selenocysteine peroxidase. These enzymes play a key role in inhibiting lipid peroxidation and protecting cells from oxidative stress [14].

According to some authors [15], pregnancy is often characterised as a state of systemic inflammation. During late gestation, rapid foetal development increases metabolic demands on the pregnant female, leading to elevated systemic oxidative stress [16,17]. This oxidative stress exacerbates the maternal systemic inflammatory response and contributes to aberrant cytokine expression, which in turn can further intensify oxidative damage [3]. It is well established that oxidative stress is a crucial factor in foetus development [18]. Consequently, any intervention that reduces maternal oxidative stress during pregnancy should positively impact foetal growth and improve the antioxidant status of newborns.

A consequence of oxidative stress can be DNA lesions, which may occur even during the foetal stage of development [19], as well as in neonatal animals due to their underdeveloped immune systems [20]. Oxidative damage is frequently implicated in dysfunction and genomic DNA damage, e.g., in the liver, leading to self-perpetuating mutations that contribute to ageing and degenerative diseases [4]. Factors released during oxidative stress, such as lipid peroxidation products, e.g., malondialdehyde (MDA), are well-established markers for assessing lipid oxidative damage in the body. MDA originates from two sources: dietary intake and peroxidation of unsaturated fatty acids in body tissues. It has cytotoxic, mutagenic and carcinogenic properties, and has long been recognised for its interactions with proteins and nucleic acids, resulting in DNA–protein crosslinks and various adducts that damage biomolecules [21], inhibit enzymes associated with cellular defence against oxidative stress [22] and those involved in the repair of altered DNA bases [23].

DNA lesions have high mutagenic potential, leading to autoimmune, inflammatory, neurodegenerative, and cardiovascular diseases; diabetes; cancer; and other age-related disorders [24]. Thus, the elimination of DNA damage is crucial for preserving genomic integrity. The main enzymes involved in the removal of damaged bases include formamidopirymidyno-DNA glikozylase (FPG) (removing 8oxoG); thymine-DNA glycosylase (TDG) (removing etenocytosine (etC)); and N-methylpurine DNA glycosylase (MPG) (removing etenoadenine (etA)) [25,26].

Long-chain n-3 PUFAs play an irreplaceable role in the proper growth and functioning of many bodily systems. Regular consumption of dietary supplements containing these fatty acids has been shown to improve cardiovascular function [27], central nervous system activity [28], and outcomes in the treatment of certain neurological and psychiatric disorders [29]. However, the literature concerning the influence of long-chain n-3 PUFAs on inflammation and oxidative processes remains inconsistent. Most studies indicate that regular intake of long-chain n-3 PUFAs improves anti-inflammatory and antioxidant mechanisms while suppressing lipid peroxidation [27]. Reported effects include increased total antioxidant capacity and GPx activity, along with reduced MDA levels [30]. These acids are also able to inhibit the production of pro-inflammatory eicosanoid [31] and pro-inflammatory cytokines [32], thereby contributing to the prevention of many chronic diseases [33]. Conversely, some studies in humans [34] and cancer cell lines [35] have reported prooxidative effects, indicating that under certain conditions, n-3 PUFAs may intensify oxidative stress.

There is abundant evidence that maternal nutrition during pregnancy and lactation is directly related to foetal and neonatal development and may have long-term consequences for postnatal growth [36]. Research indicates that maternal fatty acid nutrition can affect epigenetic modifications in the offspring and regulate gene expression in fatty acid metabolic pathways [37,38]. However, the role of long-chain n-3 PUFAs in preventing foetal and neonatal oxidative stress through maternal nutrition remains less clear. To date, the effect of maternal dietary supplementation with long-chain n-3 PUFAs on foetal oxidative status has mainly been investigated using stress markers measured in the placenta or in umbilical vein blood supplying oxygenated, nutrient-rich blood from the placenta to the liver. Therefore, the placenta, cord blood, and liver of neonatal pigs play important roles in the interplay of nutrients, oxidative stress, and inflammation between sows and their offspring. Nevertheless, research results in this area remain ambiguous, with many focusing on newborns, including the suckling period. Jones et al. [39] reported that maternal supplementation with long-chain n-3 PUFAs reduced placental oxidative stress and promoted placental and foetal growth in rats. Similar findings were presented by Opgenorth et al. [40] in calves. In contrast to the aforementioned studies, other scientists showed that while a maternal diet rich in long-chain PUFAs decreased inflammatory response in sows and their offspring, it simultaneously increased susceptibility to oxidative stress in sows and piglets [41,42,43]. Interestingly, a review of the available literature revealed a lack of data concerning the effect of maternal n-3 PUFA supplementation on oxidative stress, and the activity of DNA repair enzymes in newborn offspring before suckling. Long-chain n-3 PUFAs, such as eicosapentaenoic acid (EPA; 20:5n-3) and docosahexaenoic acid (DHA; 22:6n-3), are essential fatty acid for pigs, and have been shown to exert beneficial anti-inflammatory and antioxidant effects in animal and cell models [31,44,45]. Therefore, fish and algal oils, which are rich in EPA and DHA, could be considered as potential dietary supplements to combat oxidative stress. The present study utilised an in vivo model of neonatal piglets due to their physiological similarity to humans in terms of organ morphology and function, DNA damage repair mechanisms, and metabolic rate [46]. Moreover, neonatal piglets, like humans and other mammals, have underdeveloped immune systems and therefore are more susceptible to oxidative stress [20] and DNA damage.

Thus, the aim of the study was to determine the activity of key antioxidant defence enzymes (SOD, CAT, and GPx) and DNA glycosylases involved in base excision repair (BER) (FPG, TDG, and MPG) in the liver of newborn piglets from mothers supplemented with fish oil, algal oil or their nanoparticle forms during pregnancy. We hypothesised that (i) supplementation of pregnant gilts with fish and algal oils rich in EPA and DHA would prevent oxidative stress in their foetuses/neonates, thereby influencing the activity of DNA glycosylases responsible for repairing oxidatively damaged bases; (ii) administration of these oils in nanoparticle form would strengthen these effects due to improved absorption [23]; (iii) prevention of oxidative stress by maternal omega-3 supplementation would reduce DNA base damage, thereby lowering the activity of the BER pathway and associated repair systems such as nucleotide excision repair (NER) and mismatch repair [25,26]; nanoparticles are widely applied in biomedical and biotechnological research, including diagnostics, targeted therapies, and drug delivery systems. Their use in maternal nutrition, by improving bioavailability of fish and algal oils, was expected to provide stronger protection against oxidative stress and DNA damage in both gilts and their offspring [23,25,26].

From the perspective of socio-economic geography and spatial planning, innovations in animal nutrition, such as supplementation with long-chain n-3 polyunsaturated fatty acids (PUFAs), may have significant implications for regional food systems and public health strategies. Access to PUFA-enriched meat products remains uneven among regions, particularly between highly industrialised agricultural zones and peripheral rural areas. Such disparities contribute to territorial health inequalities [27,28]. Moreover, recent findings indicate that the consumption of omega-3-rich foods correlates positively with socio-economic status, including income and education, reinforcing geographical differences in nutritional health and related outcomes [29,30,31,32,33,34,35,36]. Addressing these inequalities requires a comprehensive approach linking nutritional innovation with regional development and public health policies.

## 2. Results

The level of oxidative stress and the activity of the antioxidant defence system in newborn piglets (on their first day of life) were modulated by supplementing pregnant gilts with fish and algae oils, administered either in natural form or as nanoparticles. Liver MDA content, and the activities of antioxidant enzymes (SOD, CAT, and GPx), and DNA repair enzymes (FPG, MPG, and TDG) were measured.

**Figure 1:**
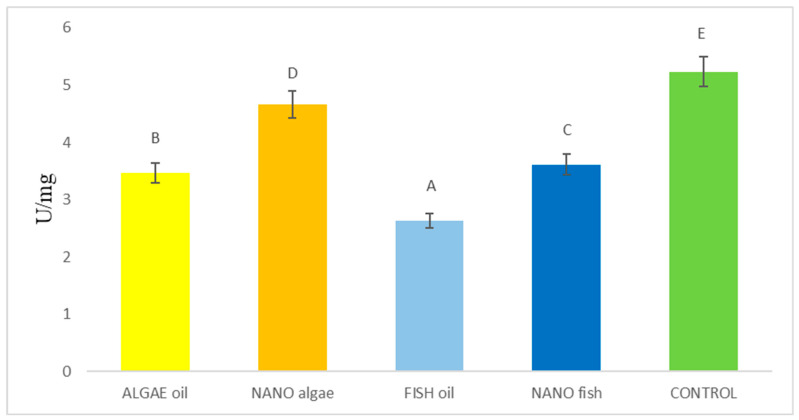
Malondialdehyde (MDA) content (µM/L) in the liver of newborn piglets (first day of life) from gilts fed diets supplemented with fish oil, algal oil, and their nanoparticles. Bars labelled with different letters (A–E) differ significantly (*p* < 0.01).

The MDA content in the liver of newborn piglets and differences between treatment groups are shown in Figure 1 and Table 1. The lowest MDA content was observed in piglets born to the fish oil-supplemented gilt (2.62 U/mg), followed by piglets born to the algal oil-supplemented gilt (3.46 U/mg), nano fish oil-supplemented gilt (3.61 U/mg), and nano algal oil-supplemented gilt (4.66 U/mg). The highest MDA content was recorded in piglets from the control gilt (5.23 U/mg) (Figure 1).

**Figure 2:**
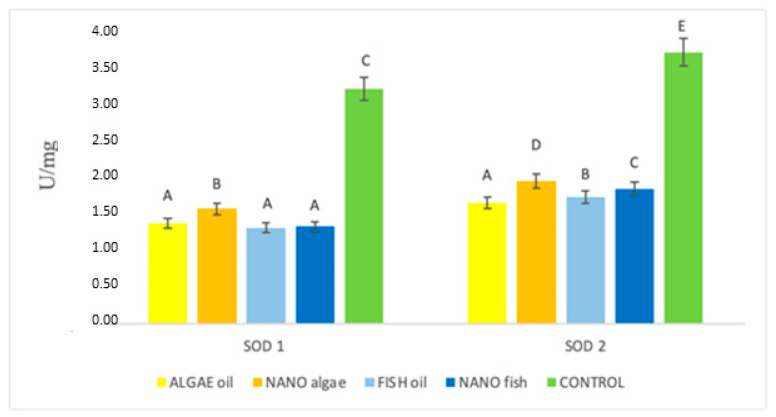
Activity (U/mg) of superoxide dismutase enzymes (SOD1 and SOD 2) in the liver of newborn piglets (first day of life) from gilts fed diets supplemented with fish oil, algal oil, and their nanoparticles. Bars labelled with different letters (A–E) differ significantly (*p* < 0.01).

The activity of SOD1 in the treatment groups is shown in Figure 2 and Table 1. The lowest activity was recorded in piglets from gilts supplemented with algal oil, fish oil, and nano fish oil (average 1.34 U/mg). Increased activity was found in piglets born to the gilt supplemented with nano algal oil (1.57 U/mg), while the highest level of activity was observed in animals born to the control gilt (3.19 U/mg). The pattern of SOD2 activity differed from that of SOD1 (*p* < 0.01). The lowest activity was observed in piglets from the gilt supplemented with algal oil (1.65 U/mg), followed by nano algal oil (1.94 U/mg), fish oil (1.73 U/mg), and nano fish oil (1.83 U/mg). The highest activity was again recorded in piglets born to the control gilt (3.74 U/mg) (Figure 2).

**Figure 3:**
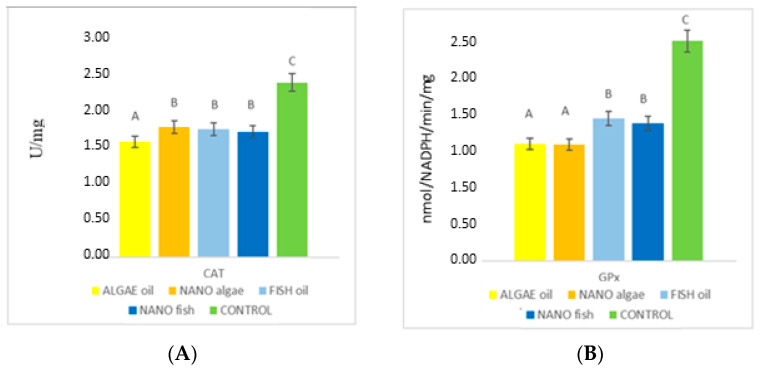
Differences in the activity (nmol/NADPH/min/mg) of catalase (CAT) (**A**) and glutathione peroxidase (GPx) (**B**) in the liver of newborn piglets (first day of life) from gilts fed diets supplemented with fish oil, algal oil, algal oil, and their nanoparticles. Bars labelled with different letters (A–C) differ significantly (*p* < 0.05).

CAT activity in the liver of newborn piglets differed between treatment groups (Figure 3, Table 1). The lowest mean activity was found in piglets born to the gilt supplemented with algal oil (1.47 U/mg), followed by fish oil, nano algal oil, and nano fish oil (average 1.63 U/mg). The highest activity was recorded in piglets from the control gilt (2.23 U/mg; *p* < 0.01; Figure 3).

**Figure 4:**
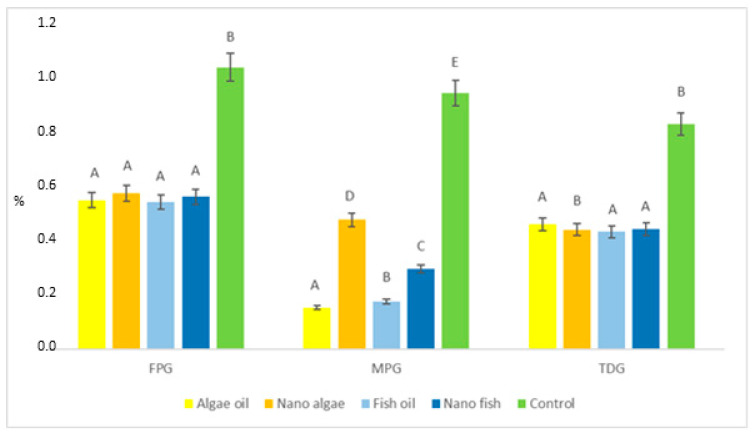
Activity of DNA repair enzymes and the proportion of modified DNA bases in the liver of newborn piglets (first day of life) from gilts fed diets supplemented with fish oil, algal oil, and their nanoparticles during pregnancy. Bars labelled with different letters (A–E) differ significantly (*p* < 0.01); FPG—formamidopirymidyno-DNA glycosylase; TDG—thymine-DNA glycosylase; MPG—N-methylpurine DNA glycosylase.

**Figure 5:**
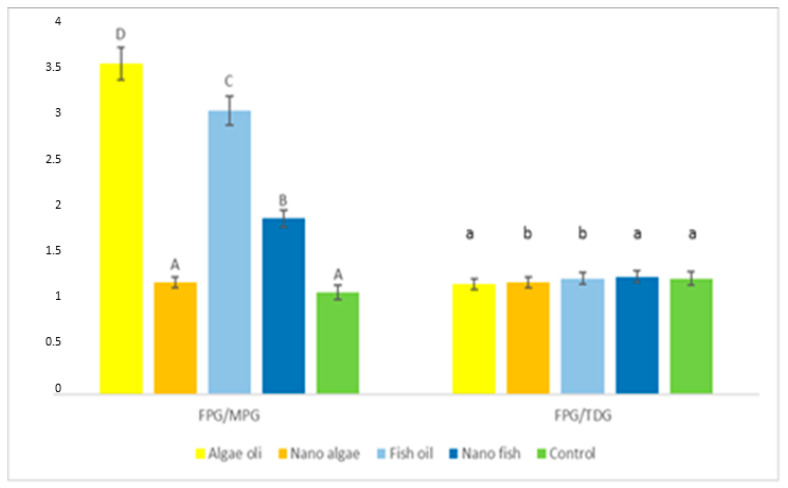
Differences in the activity of bifunctional (FPG) and monofunctional (MPG, TDG) DNA glycosylases in the liver of newborn piglets (first day of life) from gilts fed diets supplemented with fish oil, algal oil, and their nanoparticles. Bars labelled with different capital letters (A–D) differ significantly at *p* < 0.01, and bars marked with different lowercase letters (a, b) differ significantly at *p* < 0.05; MPG—N-methylpurine DNA glycosylase; TDG—thymine-DNA glycosylase.

The relationship between treatment groups for glutathione peroxidase (GPx) enzyme activity was similar to that observed for the CAT enzyme (Figure 4, Table 1). The lowest GPx activity was recorded in piglets born to gilts supplemented with both forms of algal oil (average 1.49 nmol/NADPH/min/mg); higher in piglets from gilts supplemented with both forms of fish oil (average 1.78 nmol/NADPH/min/mg); and highest in piglets from the control gilt (2.80 nmol/NADPH/min/mg) (*p* < 0.01). Genomic DNA was isolated from liver tissue and digested using the bifunctional repair glycosylase FPG and the monofunctional repair glycosylases MPG and TDG, as assessed by nicking assay and real-time PCR. The content of these enzymes in liver tissue of experimental and control piglets is presented in Figure 5 and Table 1.

The results demonstrate that oxidative DNA damage in the liver of newborn piglets, measured as oxidatively modified guanine (8-oxoG) removed by FPG, was almost two-fold lower in piglets born to gilts supplemented with either form of fish or algal oil compared to controls (0.559 vs. 1.042%; *p* < 0.01).

The activity of the MPG enzyme, which repairs oxidatively modified adenine (ethenoadenine, εA), indicated significant differences in genomic DNA damage between treatments (*p* < 0.01). The percentage of εA lesions was lowest in piglets from algal oil-supplemented gilts (0.154%), followed by fish oil (0.177%), nano fish oil (0.297%), and nano algal oil (0.478%), and highest in piglets born to the control gilt (0.947%). TDG activity, reflecting oxidatively modified cytosine (etenocytosine, etC) was similar to FPG-mediated repair, averaging 0.446% in piglets from gilts supplemented with both oils (regardless of form) and 0.832% in control piglets.

FPG activity relative to MPG, expressed as the FPG/MPG ratio (Figure 5, Table 1) was lowest (*p* < 0.01) in piglets born to gilts supplemented with nano algal oil and the control gilts (average 1.16); higher in piglets from the gilt supplemented with nano fish oil (1.90); and highest in piglets from gilts supplemented with fish and algal oils (average 3.33). No differences were observed in the activity of FPG compared to TDG (FPG/TDG ratio).

Although the core experimental results concern biochemical and physiological indicators in neonatal piglets, they have broader implications for regional food systems. Reduced oxidative stress and enhanced DNA repair in piglets from supplemented gilts imply the potential to produce meat with improved health-promoting properties. However, this potential must be considered in terms of spatial equity: regions with better-developed logistics, higher farmer awareness, and stronger institutional support are more likely to adopt such practices, while less developed areas may lag behind, exacerbating existing spatial inequalities in the production and availability of functional foods.

## 3. Discussion

### 3.1. Level of Oxidative Stress

The results of the present study clearly demonstrate that maternal supplementation of gilts with algal and fish oil reduced oxidative stress in their newborn offspring, as evidenced by the lower MDA content in liver tissue. However, the effect of algal oil supplemented in the form of nanoparticles was slightly weaker than that of natural fish oil and natural algal oil. The literature reports on the influence of n-3 PUFAs on oxidative stress are ambiguous. Several studies [43,44,45,46,47,48,49,50,51,52,53] have shown that supplementation with omega-3 LC-PUFAs during pregnancy and lactation alleviate oxidative stress in mothers, with the strongest effect recorded in neonates at birth and during the first two postnatal months. This was attributed to increased plasma total antioxidant capacity; higher levels of fat-soluble antioxidants, especially in the umbilical cord artery; and increased activity of cytosolic antioxidant enzymes (SOD and CAT) at 2.5 months of postnatal life. Tanghe et al. [43] observed a tendency toward lower MDA content in the blood plasma of piglets when gilts were fed a diet supplemented with 2% fish oil during pregnancy, although this effect was most pronounced at weaning. In contrast, Lou et al. [53,54,55,56] reported no effect of maternal fish oil supplementation on MDA levels in newborn piglets. Other authors [9] have suggested that n-3 PUFAs are susceptible to lipid peroxidation and thus could potentially exacerbate oxidative stress and tissue damage. However, other researchers [39] reported no such effect of dietary n-3 PUFAs in humans. Supplementation of pregnant women with long-chain n-3 PUFAs markedly reduced lipid peroxidation, as indicated by lower placental F2-isoprostane content, even when intake exceeded current recommendations. Although cord blood was not analysed, it is likely that oxidative stress markers were also reduced due to the protective role of the placenta [9].

### 3.2. Activity of Antioxidant Defence Enzymes

The antioxidant defence system relies on endogenous enzymatic and non-enzymatic components that act collectively against free radicals and their damaging effects on cells. Depending on their mode of action, antioxidants are classified into successive lines of defence [5]. SOD, CAT and GPx enzymes belong to the first line of defence, which plays a key role in neutralising superoxide anion radicals generated continuously as by-products of mitochondrial electron transport [4]. Numerous studies have investigated antioxidants, including long-chain n-3 PUFAs, and their role in preventing oxidative stress and associated cellular damage. However, data on the contribution of these fatty acids to oxidative stress prevention during foetal development and in newborns remain limited, particularly with respect to first-line antioxidant enzymes.

Maternal supplementation with fish and algal oils reduced liver SOD1 activity in newborn piglets from all supplemented groups. Depending on the type of oil and its form of administration, enzyme activity was 2.44 to 2.0 times lower than in the control group. Liver SOD2 activity was also decreased in piglets from gilts supplemented with fish and algal oils, regardless of the form of application. However, the activity of this enzyme showed significantly greater variation in the experimental groups. Importantly, SOD2 activity was higher in piglets whose mothers received nano-encapsulated oils compared to those receiving natural oils, with the highest activity observed in the nano algal oil group. The literature on the effects of maternal supplementation with long-chain PUFAs during pregnancy on SOD activity in newborns offspring is limited and inconsistent, and mainly focused on umbilical cord blood measurements. In human studies, no significant differences in SOD activity were found in umbilical vein or artery blood between neonates of DHA-supplemented mothers and controls [41,42,43,44,45,46,47,48,49,50,51,52,53]. Significantly higher activity of this enzyme has been reported in 2.5-month-old neonates whose mothers received DHA supplementation. Conversely, reduced SOD activity has been observed in umbilical vein blood of newborns from a mother consuming fish meat, a rich source of long-chain PUFAs, especially DHA [54]. However, contrary to our findings, the differences between treatments in the latter study were not statistically significant. Other studies indicated increased SOD activity in the brain, liver, and uterus of rats born to dams fed a diet low in omega-3 PUFAs compared to those on a DHA-rich diet [55,56,57,58,59,60,61,62,63,64,65,66]. It should be noted that these measurements were taken during postnatal development, namely five weeks after weaning. In turn, Lou et al. [56] showed that fish oil supplemented to pregnant sows had no effect on T-SOD in cord blood plasma or on SOD mRNA expression in the livers of newborn piglets, although enzyme activity was reduced in the placenta. Likewise, the offspring of sows fed n-3 PUFAs did not show evidence of altered oxidative status [43]. 

Hepatic CAT activity in newborn piglets was reduced when their mothers were supplemented with either fish or algal oil. Depending on the type and form of the oil, CAT activity was 1.34 to 1.52 times lower than in the control group. The recent literature on the effects of maternal long-chain PUFA supplementation during pregnancy on CAT activity in newborn offspring remains limited. A similar—though statistically non-significant—trend was observed by García-Rodríguez et al. [54] in the umbilical vein blood of human neonates born to mothers supplemented with DHA-rich fish [54], and no significant differences were observed in cord vein or artery blood between offspring of supplemented and non-supplemented mothers in another study [53,54,55,56,57,58,59,60,61,62,63,64,65,66,67,68,69]. In contrast, higher CAT activity has been observed in 2.5-month-old piglets born to DHA-supplemented mothers. Maternal intake of fish oil was also found to alter catalase mRNA expression in the livers of newborn piglets [56].

The current results indicate that GPx activity in the livers of newborn piglets from mothers with fish or algal oils during pregnancy (regardless of the method of administration) was downregulated. A stronger effect was observed in groups supplemented with natural algal oil and its nano-encapsulated form. Contrary to our outcomes, García-Rodríguez et al. [54] reported significantly higher GPx levels in the umbilical vein blood of neonates born to mothers supplemented with oily fish [54]. Similarly, Tanghe et al. [43] observed higher GPx enzyme activity in the liver of 5-day-old piglets from mothers supplemented with fish oil, although this effect was less pronounced in plasma, and at weaning, GPx levels in plasma and liver were comparable to controls. Meanwhile, Lou et al. [56] reported no effect of maternal fish oil supplementation on GPx in cord blood plasma or on GPx mRNA expression in the livers of newborn piglets. Tanghe et al. [43] further noted that offspring from n-3 PUFA-supplemented sows showed no overall signs of altered oxidative status. These observations align with human studies [53], which detected no significant differences in GPx activity in umbilical cord vein or artery blood following maternal DHA supplementation.

### 3.3. Activity of DNA Repair Enzymes

FPG enzyme cleaves and removes 8-oxoG DNA lesions [57], and deficiencies in this enzyme or its encoding gene (OGG1) have been linked to cellular degeneration and inflammation [58,59]. MPG primarily repairs DNA bases damaged by alkylation, such as N3-methyladenine (N3-meA), N7-methylguanine (7-meG), N3-methylguanine (N3meG), hypoxanthine, 1,N6-ethenoadenine and 8-oxoguanine (8oxoG), with higher nuclear and mitochondrial levels improving base excision repair efficiency [60]. TDG glycosylase targets DNA damage resulting from deamination, glycosylating mismatched, oxidised, or halogenated pyrimidine bases and removing 3-N(4)-ethenocytosine opposite guanine [61,62,63,64,65,66]. In the present study, the overall level of genomic DNA lesions in the liver was low, not exceeding 4%, indicating effective DNA repair activity. Nevertheless, the inclusion of fish and algal oil in the nutrition of pregnant gilts further reduced the activity of key DNA repair enzymes in the livers of their offspring. Specifically, the activity of FPG and TDG enzymes was approximately two times lower in supplemented groups compared to the control, while the reduction in MPG activity was even more pronounced. The FPG/MPG ratio indicated that lesions arising from guanine damage strongly predominated over those from adenine damage, ranging from nearly two- to over threefold depending on the treatment group. In contrast, the FPG/TDG ratio indicated that DNA lesions resulting from guanine and cytosine damage were comparable between treatment groups. The literature data indicates that the activity of glycosylases is crucial for maintaining genomic integrity in the liver [25]. While many studies on long-chain n-3 PUFAs and DNA repair involve experiments during the postnatal period, the present findings highlight the prenatal influence of these fatty acids. The observed reduction in DNA repair enzyme activity may reflect the anti-inflammatory properties of long-chain PUFAs [39]. No data are available in the literature on the effect of maternal supplementation with long-chain n-3 PUFAs on the activity of DNA base repair enzymes in the liver of their offspring. Our unpublished results indicate that feeding pregnant gilts with fish and algal oils decreased the number of damaged DNA bases (8oxo, etA and etC) in neonatal livers. In contrast, other studies reported that diets containing rapeseed or flaxseed (both rich in C18:3 n-3 acid, ALA) did not exert any significant protective effect against oxidative DNA damage in the colon of growing rats [62] or newborn piglets [64]. However, the authors of a later study [63] reported lower rates of 8oxoG lesions in the hippocampus of newborn piglets following a similar dietary treatment for pregnant sows as in the present research. These discrepancies are likely attributable to differences in the source of long-chain PUFAs (seed meal vs. natural oils and their nanoparticles) and to variations in the metabolic activity of the liver, colon, and hippocampus.

The main distinction between this study and existing literature lies in our focus on the liver as the target organ for assessing antioxidant capacity, DNA damage, and repair enzyme activity, while majority of previous studies have analysed blood or other tissues. To determine these parameters, measurements of SOD, CAT, GPx, BER pathway enzymes (FPG, MPG, TDG), and MDA, as a marker of lipid peroxidation, were selected to evaluate these aspects comprehensively. When antioxidant and DNA repair systems are not maintained at optimal levels, they can act as physiological stressors, inducing somatogenic stress. Therefore, ensuring that pregnant gilts receive an appropriate PUFA-rich diet is crucial for the development of healthy, disease-free piglets. This is particularly important given that the global population is projected to reach 9.8 billion by 2050, a 20% increase compared to today [70]. As living standards in developing countries improve, the demand for animal products is projected to increase by up to 50% [71]. In Poland, consumer habits and culinary preferences for pork result in increases in the profitability of pig production compared to other livestock species. Pork consumption is projected to rise by up to 37% by 2050 [71,72,73,74,75,76]. Future market success will become increasingly dependent on the production of lean, tender pork from farms that prioritise animal welfare and utilise advanced nutrition strategies, including feeds enriched with long-chain PUFAs. A growing body of research confirms the positive effect of these fatty acids on human health, influencing cognitive, cardiovascular, and skeletal systems during both prenatal and postnatal development [71,72,73,74,75,77,78]. Thus, modern pork production should be based not only nutritional optimisation for growth and feed efficiency, but also the beneficial properties of pork, which has significant implications for global public health.

Pig feed producers are currently facing several challenges that affect the economics of pig production. Changing market conditions, environmental regulations, and geographic factors are increasing the costs of agricultural operations. Profitability on farms can be improved through foetal programming using feeds prepared on-site, which can reduce production costs by up to 20%. However, it is especially important to balance these mixtures and supplement them with protein concentrate premixes and an appropriate content of long-chain omega-3 fatty acids. The inclusion of fats rich in n-3 long-chain fatty acids in the nutrition of pregnant sows not only benefits the health of their offspring, but also reduces production costs during early postnatal period, a phase that is particularly challenging and cost-intensive for breeders. Moreover, the results of this work could have implications for the nutrition of pregnant women, potentially with regard to improving the health of human newborns. Widespread integration of long-chain fatty acids into maternal diets could thus serve as a powerful tool in public health, bridging agricultural innovation with human well-being.

### 3.4. Mechanisms Underlying Reduced Activity of Antioxidant and DNA Repair Enzymes

The reduction of oxidative stress by long-chain n-3 PUFAs has been confirmed by decreased concentrations of malondialdehyde (MDA) and F2-isoprostanes, which are reliable markers of in vivo oxidative stress both in neonatal plasma and during early postnatal growth [65]. Evidence also suggests that a high LA–ALA ratio increases the content of TABRS, another marker of oxidative stress [66], and upregulates the expression of antioxidant enzymes [67], as arachidonic acid (AA) conversion generates pro-inflammatory mediators, whereas eicosapentaenoic acid (EPA) conversion gives rise to anti-inflammatory compounds. The beneficial effect of long-chain PUFAs on pregnancy outcome and the condition of newborns is likely mediated through a reduced oxidative burden in the placenta, as these fatty acids inhibit reactive oxygen species (ROS) production in vitro [44,68]. However, some studies reported conflicting findings that n-3 PUFA supplementation increased free radical generation and cellular damage in specific tissues like the uterus and liver [55,69]. In addition to their antioxidant effects, n-3 PUFAs regulate a number of signalling pathways involved in placental function, and thus affect foetal growth and development. These fatty acids also act as ligands for peroxisome proliferator-activated receptors (PPARs) [79]. Other authors [80] have demonstrated that all types of PPARs are expressed in the placenta and play an important role in regulating transcription factors linked to metabolic, anti-inflammatory and developmental processes in the foetus [80]. For example, PPARγ exerts anti-inflammatory effects, directly regulating the expression of inflammatory genes, and interfering with activation of the pro-inflammatory transcription factor NF-κB [31]. Supporting this, Luo et al. [41] proved that feeding pregnant sows with fish oil tended to upregulate PPARγ mRNA expression in the livers of newborn piglets, suggesting maternal supplementation with fish oil may modulate the neonatal immune system and support adaptation to the postnatal environment. Moreover, the anti-inflammatory and pro-resolving properties of n-3 PUFAs are well characterised, with documented effects on eicosanoid synthesis, production, NF- κB activation, and inflammatory cytokine expression [31]. Increased inflammation is associated with restricted foetal and placental growth [81]. Thus, n-3 PUFA supplementation and the associated increase in placental levels may promote foetal growth by limiting placental inflammation [9,54]. In addition, higher n-3 PUFA intake restricts the conversion of linoleic acid into longer-chain n-6 PUFAs (arachidonic acid) through competitive inhibition of Δ6-desaturase [82]. This reduces the generation of pro-inflammatory eicosanoids (arachidonic acid derivatives) and increases anti-inflammatory mediators (EPA derivatives), which have a positive impact on foetal development [39]. A positive effect of long-chain PUFAs on the ratio of arachidonic acid to EPA derivatives was confirmed in a study by Luo et al. [41], where fish oil supplemented to pregnant sows significantly decreased the concentration of IL-1β in cord blood plasma and tended to lower IL-1β levels in the plasma of newborn piglets. Furthermore, hepatic expression of IL-1β mRNA (pro-inflammatory) was downregulated in these piglets, while expression of IL-10 mRNA (anti-inflammatory) was upregulated. Similarly, dos Santos Silva et al. [83] reported significantly lower concentrations of pro-inflammatory markers (interleukine-2 and interleukine-6) in the blood plasma of neonatal calves (before colostrum intake) born to cows supplemented during pregnancy with n-3 PUFAs in the form of encapsulated flaxseed oil (an ALA source) and fish oil (EPA and DHA source). Furthermore, long-chain n-3 PUFAs are known to reduce inflammation through multiple mechanisms, including suppression of lipogenesis and enhanced generation of specialised mediators, such as resolvins and protectins [84] Sobrino et al. [85] demonstrated that EPA and DHA acids are also substrates in the synthesis of potent immune-protective mediators, and they actively reprogramme immune responses by regulating leukocyte activity and counteracting the production of inflammatory cytokines.

Contrary to the initial hypotheses of this study, supplementation of pregnant gilts with fish and algal oils in nanoparticle forms had a less beneficial effect on oxidative stress in their offspring compared to the natural form of these oils. This was evidenced by higher hepatic MDA levels and increased activity of antioxidant defence enzymes (particularly SOD2 and MPG). The weaker performance of nano-encapsulated forms may result from nanoparticles’ natural tendency to aggregate, which can alter their physiochemical properties, reactivity, fate, transport, and biological interactions, including their bioavailability and absorption [49,77,86,87,88,89,90,91]. Thus, contrary to common expectations, nano-encapsulation may have impeded rather than improved the placental transfer of n-3 PUFAs, limiting their availability for foetal uptake and incorporation into developing tissues. Additionally, Williams-Bey et al. [86] observed that reduced incorporation of long-chain PUFAs into phospholipid membranes diminishes their ability to inhibit the production of pro-inflammatory eicosanoids and suppresses immune cell activation and the release of pro-inflammatory cytokines. Findings from other studies have also shown that nanoparticle geometry can influence their transport across the placental barrier [87], with aggregated forms showing particularly reduced mobility and bioavailability. Herd et al. [88] have shown that nanoparticles of different geometries have very different uptake profiles at the cell surface, which in turn trigger distinct downstream cellular pathways. Even small alterations in physicochemical characteristics can markedly influence interactions of nanoparticles with the biological environment, affecting mechanisms of cellular uptake and, ultimately, intracellular fate [39,89,90]. These altered interactions can also cause unintended side effects, such as induction of the oxidation and chronic inflammatory cascades. In response, organisms may upregulate defence mechanisms, such as increasing antioxidant enzyme activity or the function of DNA repair glycosylases, to mitigate the resulting damage. Together, these factors may explain the attenuated protective effect against oxidative stress observed in the offspring of gilts receiving nano-encapsulated long-chain n-3 PUFAs. Further in-depth research is needed to confirm these mechanisms.

### 3.5. Socio–Spatial Diffusion of Nutritional Innovation

The application of n-3 PUFAs in livestock production can be regarded as a sustainable agrotechnological innovation. Its adoption and spatial diffusion depend not only on biological efficacy but also on regional infrastructure, producer knowledge, and institutional support. From a spatial planning perspective, tools such as Rogers’ diffusion of innovation theory and Geographically Weighted Regression (GWR) models offer valuable frameworks for understanding how such innovations can spread unevenly across space [70,77,86,92,93,94,95,96]. Factors such as road accessibility, agricultural extension services, and economic incentives strongly influence the rate and extent of adoption. At the same time, socio–spatial strategies promoting the consumption of PUFA-enriched meat should be embedded within local food systems (LFSs) to strengthen food sovereignty and health equity [91]. Incorporating PUFA-enhanced products into regional health and nutrition policies could serve as a preventive measure in population-level health planning, especially in underserved or nutritionally vulnerable communities.

Proper, balanced, and standardised nutrition is crucial for sow productive performance. Sows with high genetic potential and large litters can successfully raise their offspring only in optimal environmental conditions. They must receive adequate nutrition, appropriate care, maintain good health, and be housed under high-welfare conditions, which include limited stress, sufficient living space, individual housing in the farrowing room until pregnancy is confirmed, and group rearing at other times. Factors such as lighting, temperature, humidity, noise, air quality, and the spatial arrangement of sows, along with geo- and microclimatic conditions, all contribute positively to their behaviour and well-being.

The daily dose of natural oils, including their nanoparticle forms, was calculated so that each gilt received 3100 mg of DHA (600 mg for gilt and 250 mg per foetus). The activity of the SOD enzyme was generally similar across groups, although it was lower in piglets born to gilts supplemented with algal oil, fish oil and nano fish oil; higher in piglets from the gilt supplemented with nano algal oil; and highest in control animals. CAT activity followed a similar pattern, with the lowest values observed in piglets from gilts receiving algal oil and the highest in the control group. GPx activity was lowest in piglets from gilts supplemented with both forms of algal oil; higher in those from gilts receiving fish oil; and highest in the control group. Similarly, FPG enzyme activity in the liver of piglets from gilts supplemented with both fish and algal oils was almost half that of the control group. MPG activity was reduced in all supplemented groups compared with controls, and TDG activity was also lower in piglets born to gilts receiving either oil, regardless of its form. These results indicate that maternal supplementation with long-chain n-3 PUFAs modulates the antioxidant defence system and DNA repair enzyme activity in the liver of newborn piglets, with natural oils generally exerting a stronger effect than their nanoparticle counterparts.

The geographical implications of this study highlight the importance of regionalised food policy strategies that address spatial health inequalities and ensure equitable access to functional foods, such as PUFA-enriched pork [97]. Promoting such products within public health and nutrition frameworks can help mitigate socio-territorial disparities. However, successful implementation depends on regional variability in adoption capacity, including technological, infrastructural, and socio-economic factors [97]. Therefore, the evaluation of nutritional interventions in animal production should consider not only physiological outcomes, but also their spatial accessibility and regional transfer potential.

## 4. Materials and Methods

### 4.1. Ethics

All procedures were conducted in accordance with Polish Animal Protection Act and were approved by the II Local Ethics Committee on Animal Experimentation of Warsaw University of Life Sciences, SGGW, Warsaw, Poland (Resolution WAW2/040/2023) from 19 April 2023. The experiment was designed to minimise the number of animals whilst maintaining high statistical power according to the principles of the 3Rs (replacement, reduction, and refinement).

### 4.2. Animals’ Housing and Feeding

The study focused on the offspring of five gilts (first pregnancy). Pregnant gilts (Polish Landrace) were purchased from the Animal Breeding Centre in Chodeczek, Poland. After pregnancy was confirmed, the animals were transported to the Laboratory of Large Animal Model of the Kielanowski Institute of Animal Physiology and Nutrition, Polish Academy of Science. The gilts were transported from the breeding farm to the experimental pigsty at the Institute of Animal Physiology and Nutrition of the Polish Academy of Sciences in Jabłonna after pregnancy which was confirmed by a veterinarian (at 5 weeks of gestation). The animals were transported in specially adapted vehicles in accordance with applicable Polish law.

After an adaptation period, gilts were fed a standard diet with a nutritional value adapted to their physiological condition (i.e., early pregnancy or late pregnancy) [7]. The diets (both for early and later-term pregnant gilts) were balanced in terms of energy, protein, and amino acids and vitamins. The manufacturer’s guaranteed nutrient content is standard. This is a standard feed mixture used in sow nutrition. The nutritional and energetic values of the diet used are presented in Table 2.

After confirmation of the pregnancy, each gilt received a different fat supplement: fish oil, algae oil, fish oil nanoparticles, or algae oil nanoparticles. One gilt did not receive any fat supplements containing long-chain fatty acids and was considered as the control gilt. Fish oil and algae oil (in both forms) were added individually to the feed just before feeding. Due to the lack of dietary recommendations for pregnant sows regarding the daily intake of docosahexaenoic acid (C22:6 n-3, DHA), its amount was determined based on the recommendations for pregnant women. Thus, daily dose of natural oils, also in the form of nanoparticles of these oils, was calculated so that each gilt received a DHA dose of 3100 mg (600 mg for gilt and 250 mg for each foetus × 10 = 2500 mg). The number of developing foetuses was estimated at 10. Nanoparticles of fish and algal oils were prepared according to the method described by [23]. The experimental factor was dosed daily individually for each gilt. A precisely measured dose of oils (both forms: natural oils and nanoparticles) was added to the daily feed ration. This feeding method allowed for precise dosing of feed intake and the experimental factors. Throughout the entire study period, no uneaten feed was observed, which means that the planned/dosed amount of oils was consumed in its entirety.

The fish and algal oils used in the study were purchased from NORSAN Poland LLC, a certified producer of oils from arctic cod and algae. The composition of the oils is given in Table 3.

Till day 90 of pregnancy, the gilts were kept in individual pens (2.75 m^2^), while from day 90 onwards they were kept in a farrowing pen (5.5 m^2^). Each pen (both individual and farrowing) was equipped with a rubber mat, a feeder, and a nipple drinker. Central heating and air conditioning systems (Fancom, model ISM0.12; Fancom BV, Wilhelminastraat 17, 5981 XW Panningen, The Netherlands) in the piggery made it possible to keep the animals under thermo-neutral conditions according to [7]. The piglets were euthanized in the first 24 h after birth using Exagon 400mg/mL including sodium pentobarbital. Pentobarbital was administered into the marginal ear vein (0.1 mL/kg body weight). Dilution with sterile, isotonic NaCl solution (0.9%) in a ratio of 1:1 was necessary. After euthanasia, a sample of the liver was collected from each piglet. Because the number of piglets in the litter turned out to be smaller than expected, liver samples were taken from only six piglets from each sow. After the euthanasia of the piglets, the sows were slaughtered in the experimental slaughterhouse of The Kielanowski Institute of Animal Physiology and Nutrition Polish Academy of Science. Before slaughter, the pigs were electrically stunned (STZ 3 apparatus; P.P.H. MASTER Sp. J., Solec Kujawski, Poland).

### 4.3. Measurement of the Activity of Antioxidant Defence Enzymes

All proteins were isolated by RIPA lysis buffer according to protocol [46].

SOD activity was determined spectrophotometrically in a 48-well plate by the method described by Fridovich [11]. Protein activity was analysed in each well of the plate by adding a total volume of 100 μL of solution: 50 μL bacterial strain K12, R2, R3 or R4 modified by one of the nineteen compounds in Tryptone Soya Broth (TSB) medium which was inoculated with 2 × 10^6^ colony-forming units (CFU)/mL (approximately 0.5 McFarland units) of the bacterial strains and 50 μL component from the set number CS0009 recommended for SOD determination by Sigma Aldrich Darmstadt, Darmstadt (Germany), according to the manufacturer’s instructions. The total enzymatic activity of SOD involves the generation of superoxide anion in the “xanthine–xanthine oxidase system” with NBT as an O_2_ detector. The principle of the method is based on two reactions. The first involves the generation of a superoxide radical by the action of xanthine oxidase on xanthine. Then, NBT reacts with the superoxide radical to form a dark blue formazan dye, the growth rate of which is recorded spectrophotometrically at 540 nm. A unit of SOD activity is defined as the amount of enzyme that reduces the rate of the NBT to formazan conversion reaction by 50%, and the change in absorbance is 0.020 units/min.

CAT activity was determined spectrophotometrically in a 48-well plate by the method outlined by Aebi [47]. Protein activity was analysed in each well of the plate by adding a total volume of 100 μL of solution: 50 μL bacterial strain K12, R2, R3 or R4 modified by one of the nineteen compounds in Tryptone Soya Broth (TSB) medium which was inoculated with 2 × 10^6^ colony-forming units (CFU)/mL (approximately 0.5 McFarland units) of the bacterial strains and 50 μL buffer, according to Venisse et al. [48], including 0.01 (*v*/*v*) Triton X-100, 50mM potassium phosphate buffer (pH 7.0) or sodium phosphate buffer (pH 7.0), respectively containing 1 mM polyethylene glycol, 1mM phenylmethylsulphonyl fluoride, 8% (*w*/*v*) to a final concentration 0.1% and H_2_O_2_ to a final concentration of 9.8 mM. All measurements were made according to instructions [7,49]. A unit of CAT activity was defined as the amount of enzyme required for the degradation of H_2_O_2_ within 2 min at 240 nm per mg of protein (catalase) and after visually observing the bubbles which formed after the reaction (after adding hydrogen peroxide).

The total enzymatic activity of GPx was determined using the method of Hopkins and Tudhope [50], and involved the measurement of NADPH oxidation coupled with glutathione disulfide reduction by glutathione reductase. Changes in NADPH concentration were measured at 340 nm for 5 min. Enzyme activity was expressed as μM of oxidized NADPH2 per μg of protein. Therefore, the total protein concentration in tissue homogenates was analysed spectrophotometrically using the Bradford method with the Bio-Rad Protein Assay Kit II (Bio-Rad, Hercules, CA, USA), according to the manufacturer’s instructions.

Determinations of all three enzymatic activities were performed separately using the Freedom EVO^®^ series pipetting station (Tecan, Mannedorf, Switzerland), a modular system with an incubator, a shaker, and a sample plate reader, depending on the application and required throughput. The operation of the Freedom Evo system is based on the Evo software version 2.8 SP7, which enables control of the entire process carried out at the workstation. The assays were repeated three times. Additionally, total protein concentration in bacterial tissue homogenates was analysed spectrophotometrically by the Bradford method using the Bio-Rad Protein Assay Kit II (Bio-Rad, Hercules, CA, USA), according to the manufacturer’s instructions. All enzyme activities, determining the rate of conversion of substrate into product, are expressed in relative units per microgram of protein per unit of time (min).

### 4.4. Measurement of the Activity of DNA Repair Enzymes: Formamidopirymidyno-DNA Glycosylase (FGP), Thymine-DNA Glycosylase (TDG), and N-Methylpurine DNA Glycosylase (MPG) Was Described in Detail in the Work by Kowalczyk [25]

The genomic DNA from all liver samples was isolated from 2ml of fresh medium by using a New England Biolabs Kit (Labjot, Warsaw, Poland) according to the manufacturer’s instructions. The obtained DNA was digested by Fpg protein according to the manufacturer’s instructions (New England Biolabs, Ipswich, MA, USA cat no. M0240S, 8000 U/mL) as follows: Fpg protein was diluted 50-fold with 10× NEB buffer (provided by the Fpg protein manufacturer) and mixed with 100× BSA solution (also supplied with Fpg protein). Next, 8 µL of purified genomic DNA was mixed with 2 µL of Fpg solution and 2 µL of NEB buffer and incubated at 37 °C for 30 min. Control bacterial DNA (incubation without the tested compounds) and digested and undigested genomic DNA (incubated with the analysed compounds) samples were evaluated by 1% agarose gel electrophoresis. The DNA concentration was determined spectrophotometrically from the A_260_/A_280_ ratio. The level of oxidative damage was estimated using Image Quant version 5.2. software.

The use of Fpg protein known as 8-oxoguanine DNA glycosylase or (formamidopyrimidine [fapy]-DNA glycosylase) is important because it removes a broad spectrum of oxidized and alkylated bases from double-stranded DNA: 7, 8-dihydro-8-oxoguanine (8-oxoguanine), 8-oxoadenine, unsubstituted and substituted imidazole ring-opened purines introduced into DNA by hydroxyl radicals (e.g., FapyG, FapyA), as well as by chemical carcinogens, including anticancer drugs (e.g., Fapy-7MeG, Fapy-7EtG, Fapy-7aminoethylG, aflatoxin B_1_-fapy-guanine, 5-hydroxy-cytosine, and 5-hydroxy-uracil) [25]. The Fpg protein has two additional activities: (i) AP-lyase activity, which cleaves both 3’ and 5’ to the AP site, thereby removing the AP site and leaving a 1 base gap by alpha–beta-elimination, and (ii) dRPase activity which removes the 5′ terminal deoxyribose phosphate from DNA incised by an AP endonuclease [25].

The results were estimated based on a comparison of samples digested and non-digested with Fpg protein. The percentage of cleavage was determined based on the change in forms resulting from the enzymatic activity of the digested sample, relative to the DNA-binding Fpg protein. From the literature data, it is known that the degree of genomic DNA damage above three percent by Fpg protein indicates strong genomic DNA damage to all oxidized and modified guanines occurring as 8-oxoguanine, fapy-adenine (FapyG), and fapy-guanine (FapyA).

### 4.5. Measurement of Malondialdehyde (MDA)

The measurement method was described in detail by Bird and Draper [51] and Yilgor and Demir [52].

A commercially available MDA solution (Merk) was used and serially diluted in purified water to prepare the standard curve. The concentrations of 50–25–12.5–5–2.5–1.25–0.625 nmol/mL were used. To 25 μL of each standard or sample, 500 μL of the 2 M AcONa Buffer pH 3.5 + 0.2% TBA was added and incubated at 95 °C for 1 h. At this stage, lipoperoxides underwent hydrolysis, releasing MDA molecules, which then bound with two TBA molecules to form the MDA-TBA_2_ adduct. At the end of this stage, standards and samples were kept on ice. Next, 500 μL of the 50 mM KH_2_PO_4_ Buffer pH 6.8 was added to each standard and sample and then mixed by vortexing. This step is critical because the MDA-TBA_2_ adduct is unstable at this pH, so the manipulator must work fast. Finally, all the tubes were centrifuged at 1000 g for 5 min at 4 °C.

Then, 200 μL of the supernatant was pipetted into a new tube, and 200 μL of the 50 mM KH_2_PO_4_ Buffer pH 3.5 was added and mixed by vortexing. Finally, the 200 μL was aliquoted in a new tube for further analyses, then 800 mL of phosphate buffer, 25 mL of butylated hydroxy toluene (BHT) solution, and 500 mL of 30% tricarboxylic acid (TCA) were added to it. The tubes were mixed by vortexing and kept on ice for 2 h. The mixture was then centrifuged at 2000 rpm for 15 min. After that, 1 mL of the obtained supernatant was taken and transferred to another tube. Then 75 mL of ethylenediamine tetra acetic acid (EDTA) and 250 mL of thiobarbutiric acid (TBA) were added to this mixture. The tubes were mixed by vortexing again and kept in a hot water bath for 15 min. The tubes were then brought to room temperature. Absorbance values were read in the spectrophotometer at 532 nm.

The level of malondialdehyde was calculated as follows:

C = F × 6.41 × A. C: Concentration, F: Dilution factor, A: Absorbance

### 4.6. Statistical Analysis

Statistical analyses were performed using Statistica (version 13.1, StatSoft, Tulsa, OK, USA). The examined characteristics in different groups are presented as mean values. All the results were analysed with one-way ANOVA. When the F ratio was significant, Tukey’s honest significant difference post hoc analysis was performed. Statistical significance was set at *p* < 0.05. A borderline significant trend was set at *p* < 0.1.

### 4.7. Spatial Embedding of the Experiment

The experimental phase of this study was conducted in the Kuyavian-Pomeranian Voivodeship (north-central Poland), a region characterized by a moderate level of agricultural industrialization and a significant share of pig farming. The economic structure and location of the farms provide a relevant spatial framework for assessing the transferability of dietary innovations across diverse agricultural regions in Poland. From a spatial modelling perspective, the regional deployment of PUFA-based supplementation in animal husbandry could be optimized using GIS tools and spatial clustering techniques, enabling the identification of priority zones for technological diffusion and implementation [91,93]. Additionally, these tools can help map “nutritional deserts”—areas with limited access to nutritionally beneficial products—and guide future policy interventions.

## 5. Conclusions

The results of the present study indicate that supplementation of pregnant gilts with algal and fish oils, whether in natural or nanoparticle form, effectively reduces oxidative stress and associated DNA damage in their newborn offspring. Consequently, the activity of the antioxidant system, assessed through the levels and activity of antioxidant and DNA repair enzymes, is lower. The findings also reveal that oils administered in nanoparticle form were less effective than their natural counterparts.

Further research using molecular approaches in animal models of programmed nutrition will enable the development of targeted nutritional strategies with direct implications for humans. Inadequate energy balance or insufficient intake of nutrients with an appropriate energy value can significantly affect foetal development. Nutritional programming for humans links maternal diet during pregnancy with the future health and quality of life of the child. A well-balanced diet can reduce the risk of diseases during pregnancy and support optimal child development. The aim of this research is to guide the development of nutritional programmes that support optimal foetal growth while delivering significant health-promoting benefits.

Further interdisciplinary research is required to explore how territorial factors influence the adoption of nutritional innovations in agriculture. We propose mapping regional readiness for adopting PUFA-enriched feed using spatial indicators, e.g., farm density, infrastructure, and access to veterinary services; assessing consumer access to PUFA-rich meat products through analyses of food deserts and socio-demographic data; integrating spatial health indicators with supply chain modelling to guide evidence-based policymaking in food and health sectors; and incorporating spatial equity measures into nutritional programming to ensure that foetal health benefits from maternal dietary interventions are accessible across all regions.

This geographical perspective complements the biological findings of the study and provides a roadmap for equitable innovation in sustainable animal production and public health nutrition.

## Figures and Tables

**Table 1:** Statistical significance of differences in the activity of antioxidant enzymes between treatment groups.

Antioxidant Enzymes	Algal Oil	Nano Algal	Fish Oil	Nano Fish	Control	SE Pooled
SOD1 (U/mg)	1.37 A	1.57 B	1.31 A	1.33 A	3.19 C	0.038
SOD2 (U/mg)	1.65 A	1.94 D	1.73 B	1.83 C	3.74 E	0.0171
CAT (U/mg)	1.47 A	1.66 B	1.63 B	1.60 B	2.23 C	0.017
GPx (nmol/NADPH/min/mg)	1.49 A	1.48 A	1.81 B	1.75 B	2.80 C	0.031
DNA glycosylases						
FPG (fmol/µg of protein/h)	0.551 A	0.577 A	0.544 A	0.564 A	1.042 B	0.0089
MPG (fmol/µg of protein/h)	0.154 A	0.478 D	0.177 B	0.297 C	0.947 E	0.0038
TDG (fmol/µg of protein/h)	0.462 A	0.442 A	0.434 A	0.444 A	0.832 B	0.0114
FPG/MPG	3.58	1.21 A	3.07 C	1.90 B	1.10 A	0.0326
FPG/TDG	1.19	1.31	1.25	1.27	1.25	0.0138
MDA (µg/L) (lipid peroxidation)	3.46 B	4.66 D	2.62 A	3.61 C	5.23 E	0.0179

SE—standard error; SOD1 and SOD2—superoxide dismutases; CAT—catalase; GPx—glutathione eproxidase; MDA—malondialdehyde. Means within a row with different letters (A, B, C, D, E) differ significantly (*p* < 0.01); FPG—formamidopirymidyno-DNA glycosylase; TDG—thymine-DNA glycosylase; MPG—N-methylpurine DNA glycosylase.

**Table 2:** The standard diet with nutritional value adapted to the physiological condition of gilts.

Item	Day of Pregnancy	
	Till 90	90–114
Nutritional value (per kg diet)		
Crude protein, g	145	175
Lysine, g	8.0	10.0
Methionine	3.0	3.0
Methionine + Cystine	6.0	7.0
Threonine, g	6.0	6.5
Tryptophan, g	2.0	2.0
Crude fibre	50.0	36.0
Calcium, g	8.0	9.0
Total phosphorus	6.0	6.5
Digestible phosphorus, g	2.0	3.0
Vitamin A, I.U	13,000	12,500
Vitamin E, mg	100	100
Vitamin D, I.U.	2000	2000
Metabolizable energy, MJ	12.0	13.0

**Table 3:** Fatty acid content in the fish and algal oils used in the study.

Ingredient	Norsan Omega-3 Arktis (Per 10 mL)	Norsan Omega-3 Vegan (Per 10 mL)
Components		
Sunflower oil	-	-
Fish oil	8.8 g	-
Algal oil	-	7.0 g
Olive oil	-	2.2 g
Fatty acids’ composition		
SFA	1.8 g	-
MUFA	3.9 g	-
PUFA	2.3 g	4.4 g
Omega-3	2.0 g	4.0 g
EPA	750 mg	1218 mg
DPA	95 mg	314 mg
DHA	975 mg	2316 mg

## Data Availability

The data presented in this study are available on request from the corresponding author.

## Abbreviations

SOD	Superoxide dismutase.
CAT	Catalase.
GPx	Glutathione peroxidase.
FPG	Formamidopirymidyno DNA glycosylase.
MPG-N	Methylpurine DNA glycosylase.
TDG	Thymine-DNA glycosylase.
BER	Base Excision Repair.
PUFA	Polyunsaturated Fatty Acid.
OGG1-8	Oxoguanine glycosylase.
ROS	Reactive Oxygen Species.
RNS	Reactive nitrogen Species.
EPA	Eicosapentaenoic acid.
DHA	Docosahexaenoic acid.
(TAC)	Antioxidant capacity
MDA	Malondialdehyde.
PGE2	Pro-inflammatory prostaglandins.
PGE3	Anti-inflammatory properties.
(εA)-1,N^6^	Ethenoadenine.
(εC)-3,N^4^	Ethenocytosine.
(εG)-N^2^,3	Ethenoguanine.
(εG)-1,N^2^	Ethenoguanine.
8oxoG	8-Oxoguanine.
Fpg	Formamidopirymidyno-DNA glycosylase.
LA	Linoleic acid.
ALA	Alpha lipoic.
AA	Arachidonic acid.
TABRS	Thiobarbituric Acid-Reactive Substances.
